# Exploitation of a shared genetic signature between obesity and endometrioid endometrial cancer

**DOI:** 10.3389/fsurg.2023.1097642

**Published:** 2023-01-24

**Authors:** Junyi Duan, Jiahong Yi, Yun Wang

**Affiliations:** ^1^ First Clinical Medical College, Shanxi Medical University, Taiyuan, China; ^2^ Sun Yat-Sen University Cancer Center, Sun Yat-Sen University, Guangzhou, China; ^3^ Department of Obstetrics and Gynecology, The 985th Hospital of The People's Liberation Army Joint Logistic Support Force, Taiyuan, China

**Keywords:** endometrioid endometrial cancer, obesity-related genes, weighted gene coexpression network analysis, immune correlation analyses, targeted treatment

## Abstract

**Aims:**

The findings in epidemiological studies suggest that endometrioid endometrial cancer (EEC) is associated with obesity. However, evidence from gene expression data for the relationship between the two is still lacking. The purpose of this study was to explore the merits of establishing an obesity-related genes (ORGs) signature in the treatment and the prognostic assessment of EEC.

**Methods:**

Microarray data from GSE112307 were utilized to identify ORGs by using weighted gene co-expression network analysis. Based on the sequencing data from TCGA, we established the prognostic ORGs signature, confirmed its value as an independent risk factor, and constructed a nomogram. We further investigated the association between grouping based on ORGs signature and clinicopathological characteristics, immune infiltration, tumor mutation burden and drug sensitivity.

**Results:**

A total of 10 ORGs were identified as key genes for the construction of the signature. According to the ORGs score computed from the signature, EEC patients were divided into high and low-scoring groups. Overall survival (OS) was shorter in EEC patients in the high-scoring group compared with the low-scoring group (*P* < 0.001). The results of the Cox regression analysis showed that ORGs score was an independent risk factor for OS in EEC patients (HR = 1.017, 95% confidence interval = 1.011–1.023; *P* < 0.001). We further revealed significant disparities between scoring groups in terms of clinical characteristics, tumor immune cell infiltration, and tumor mutation burden. Patients in the low-scoring group may be potential beneficiaries of immunotherapy and targeted therapies.

**Conclusions:**

The ORGs signature established in this study has promising prognostic predictive power and may be a useful tool for the selection of EEC patients who benefit from immunotherapy and targeted therapies.

## Introduction

Endometrial cancer (EC) is the most prevalent tumor in the female genital system, and its morbidity and mortality are gradually increasing (1). Despite significant advances in various aspects of EC management, the accumulating disease burden of EC has not been reversed. Molecular typing based on genomic features has deepened our understanding of EC and as a result, clinical practice has changed as a result. It is critical to further analyze The Cancer Genome Atlas (TCGA) data to improve our understanding of EC and to address rising morbidity and mortality (2).

Obesity is a growing hazard attracting tremendous attention. Meanwhile, its association with EC and the impact of weight loss on the prevention and prognosis of EC have been the focus of gynecologic oncologists and patients (3). The results of traditional observational studies and Mendelian randomization analyses showed that the risk of EC increases with increasing Body Mass Index (BMI) and was much more relevant than other tumors. Correspondingly, bariatric surgery was effective in reducing the risk of EC (4–6). Epidemiological data demonstrated a correlation between obesity and EC, but evidence from transcription profiling is still inadequate.

This study focused on endometrioid EC (EEC), the pathological subtype that is more strongly associated with obesity (7). Obesity-related genes (ORGs) were identified by microarray data from obese women, and subsequently the ORGs signature was established in EEC patients. We analyzed the ORGs scoring groups in terms of clinical characteristics, immune function, and drug sensitivity. Through this study, we hope to better understand the potential mechanisms of EEC and obesity, find novel biomarkers that can be applied for screening and treatment, delineate subgroups to seek potential beneficiaries of targeted therapy, and take a step further toward precision medicine for EEC.

## Methods

### Data source

Series GSE112307 from the Gene Expression Omnibus (GEO) database was utilized to identify ORGs (8). Microarray data from GSE112307 were derived from 54 paired subcutaneous adipose tissues from 27 moderately obese women, collected before or after a calorie-restricted diet. The GEOquery and illuminaHumanv3.db R packages were applied for data download and gene annotation. RNA sequencing, tumor somatic mutation and clinical data from EEC patients were manually downloaded from the TCGA portal. TPM data were extracted from the RNA sequencing data for signature construction and functional analyses, and the maftools and XML R packages were used to collate mutation and survival data.

### Identification of ORGs

Weighted gene co-expression network analysis (WGCNA) is an algorithm that explores the relationship between expression and phenotype data based on correlation coefficients (9). In this study, WGCNA was used to identify gene modules associated with obesity and was implemented using the limma and WGCNA R packages. First, we performed sample clustering and removed abnormal samples. The correlation between genes was calculated. The appropriate *β* was then selected based on correlation coefficients to build the matrix and evaluate the correlation of gene expression patterns. On the basis of this, gene hierarchical and module clustering was performed to determine the correlation between gene modules and obesity based on the eigenvalues of the gene modules and the obesity or not of the samples. We then performed functional enrichment analysis and visualization of gene modules highly associated with obesity, using the clusterProfiler, org.Hs.eg.db and enrichplot R packages.

### Training and testing of ORGs signature

The samples of EEC are divided, half for the training cohort and half for the test cohort. ORGs associated with the prognosis of EEC patients were screened by univariate Cox regression in the training cohort. Subsequently, the least absolute shrinkage and selection operator (LASSO) regression was applied to further select variables and avoid model overfitting. Finally, a prognostic ORGs signature was established using stepwise multivariate Cox regression. ORGs score was calculated for each sample according to the following equation: $\mathrm{ORGs}\;\mathrm{score}=\sum_{i}\;\mathrm{coefficient}\left(\mathrm{ORG}s_{i}\right)\times \mathrm{expression}\left(\mathrm{ORG}s_{i}\right)$. The differences between the ORGs scoring groups were assessed by scatter plots and principal component analysis (PCA). The log-rank test was performed to compare overall survival (OS) between the two groups. The above analyses were validated in the test cohort and in the entire cohort. The survminer, survival, glmnet, ggplot2, pheatmap, and scatterplot3d R packages were utilized for training and testing of the ORGs signature.

### Correlation analysis of clinical features

We performed Cox regression analysis to elucidate whether ORGs score was independent of other relatively complete clinicopathological characteristics (age, race, FIGO stage and tumor grade) and visualized as forest plots. In addition, we examined the predictive power of ORGs signature in different clinical subgroups. We integrated the available clinical information to develop a nomogram using regplot, rms and survivor R packages. The accuracy of the nomogram was assessed using calibration curves.

### Correlation analysis of immune function

Single-sample gene set enrichment analysis (ssGSEA) was used to assess the infiltration and function of immune cells. We then investigated the differences in immune checkpoint gene expression between ORGs groups. Immune and stromal infiltration in the tumor microenvironment was evaluated in two groups based on the ESTIMATE algorithm (10).

### Gene mutation analysis

Somatic mutation data from TCGA were collated and analyzed using the maftools R package. The 15 genes with the highest tumor mutation frequency (TMF) in each ORGs group were visualized by waterfall plots. The tumor mutation burden (TMB) was calculated for each sample. After establishing subgroups based on median TMB, we compared the survival differences between TMB groups and confirmed the prognostic value of ORGs groups in TMB subgroups. The cBioPortalData R package was applied to download data about the microsatellite instability (MSI) status of EEC patients. Whereafter, differences in MSI status in ORGs groups, differences in ORGs score in MSI subgroups, and differences in prognosis were analyzed.

### Drug sensitive analysis

The oncopredict R package was implemented to predict drug response in ORGs groups (11). Half maximal inhibitory concentrations (IC50) of antitumor drugs were calculated based on data from the Genomics of Drug Sensitivity in Cancer (12).

### Statistical analysis

The entire analysis was implemented using R (version 4.0.3). Cox regression and survival analyses were performed by the survivor and survminer R packages. The pheatmap R package was used to draw heat maps. Wilcoxon rank sum test was applied to test the discrepancies between quantitative data. We applied Pearson correlation analysis to calculate the correlation coefficients. *P* < 0.05 was considered statistically significant.

## Results

### Identification and functional annotation of obesity-related genes

**Figure 1:**
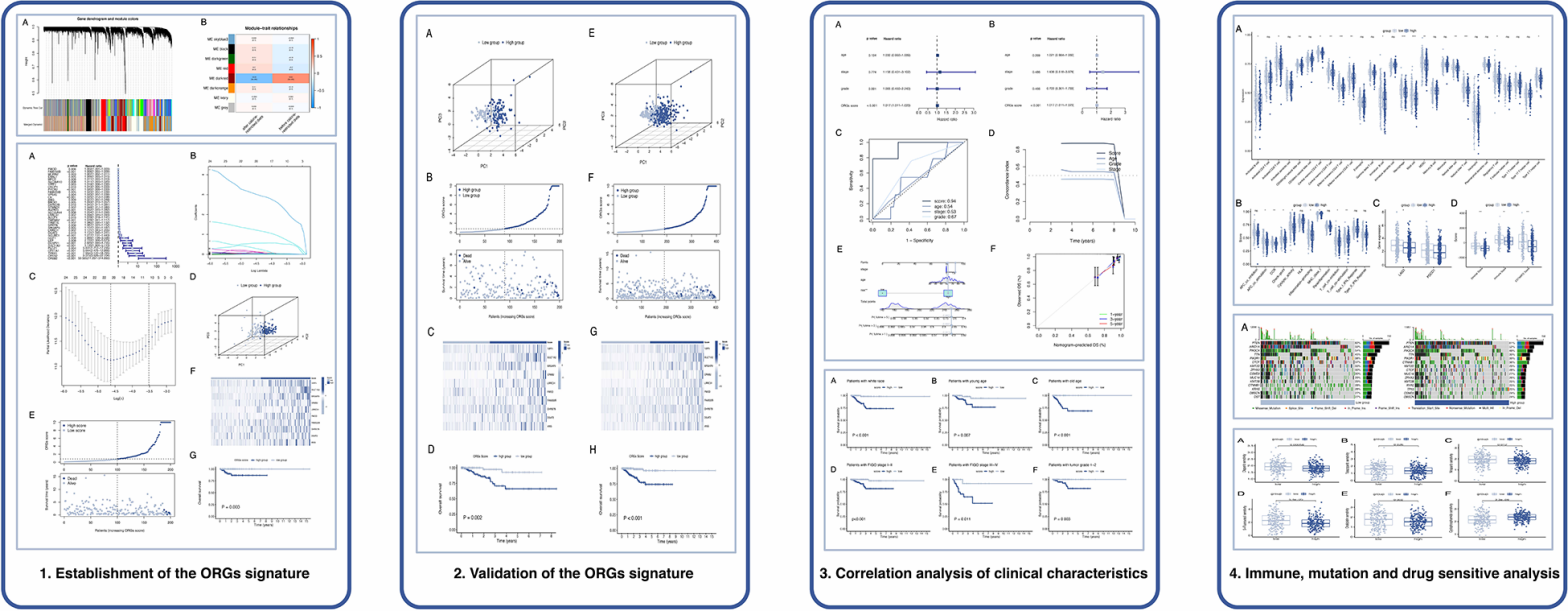
The flow plot of this study.

**Figure 2:**
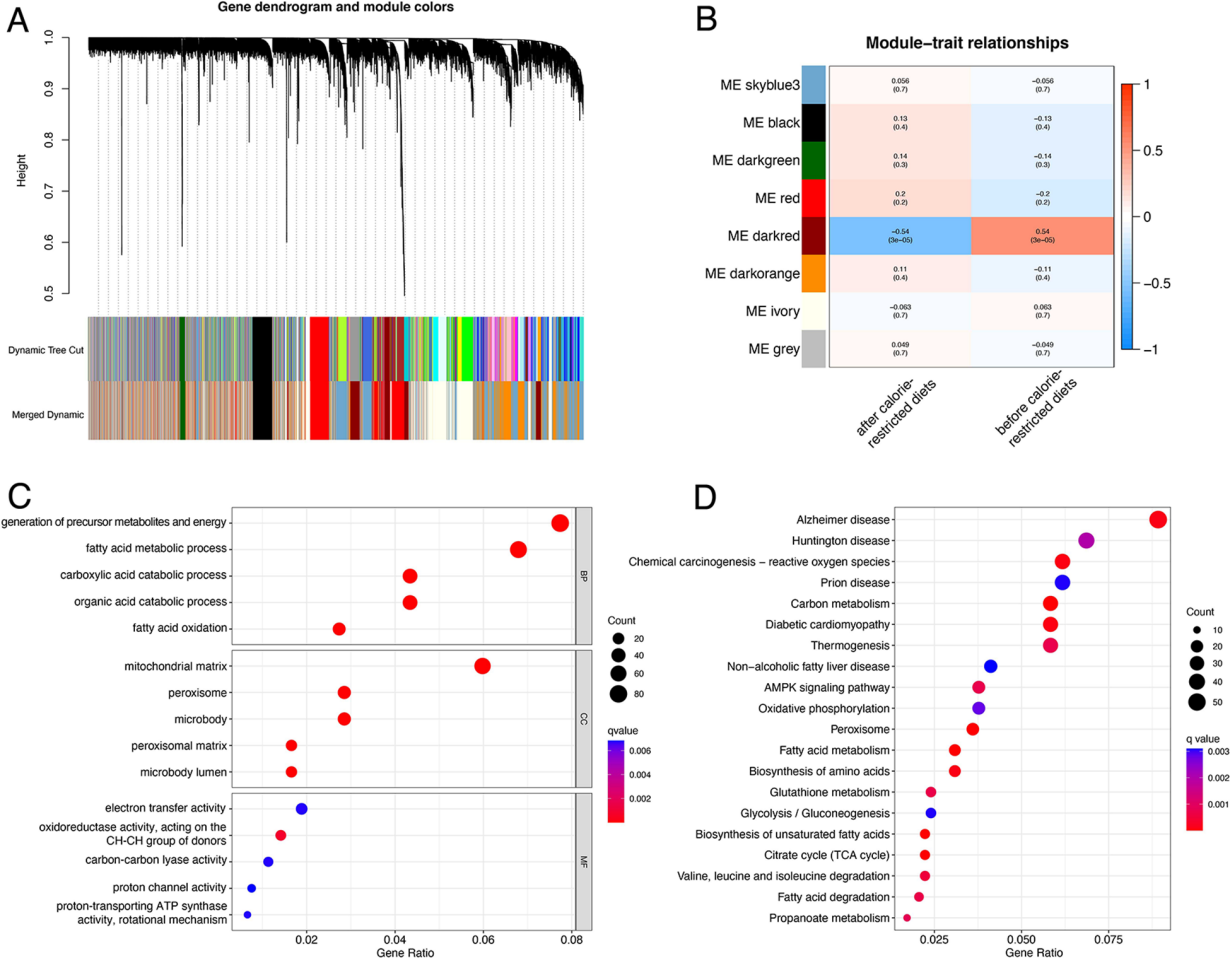
Identification and functional annotation of ORGs. (**A**) The cluster dendrogram of ORGs in obesity. (**B**) Correlation of WGCNA modules and obesity. (**C**) GO analysis of ORGs. (**D**) KEGG analysis of ORGs. ORGs, obesity-related genes. WGCNA, weighted gene co-expression network analysis. GO, Gene Ontology. KEGG, Kyoto Encyclopedia of Genes and Genomes.

The flow plot of this study was summarized in Figure 1. A gene clustering dendrogram was generated based on the GSE112307 dataset using WGCNA (Figure 2A). The expression matrix was divided into six gene modules, and the dark red module containing 1,148 genes was significantly associated with obesity and identified as ORGs (Figure 2B). The results of enrichment analysis showed that ORGs were significantly enriched in pathways related to fatty acid metabolism and biological oxidation, and were involved in transmembrane transport of substances in the form of enzymes and transporters (Figures 2C,D).

### Training and testing of the ORGs signature

**Figure 3:**
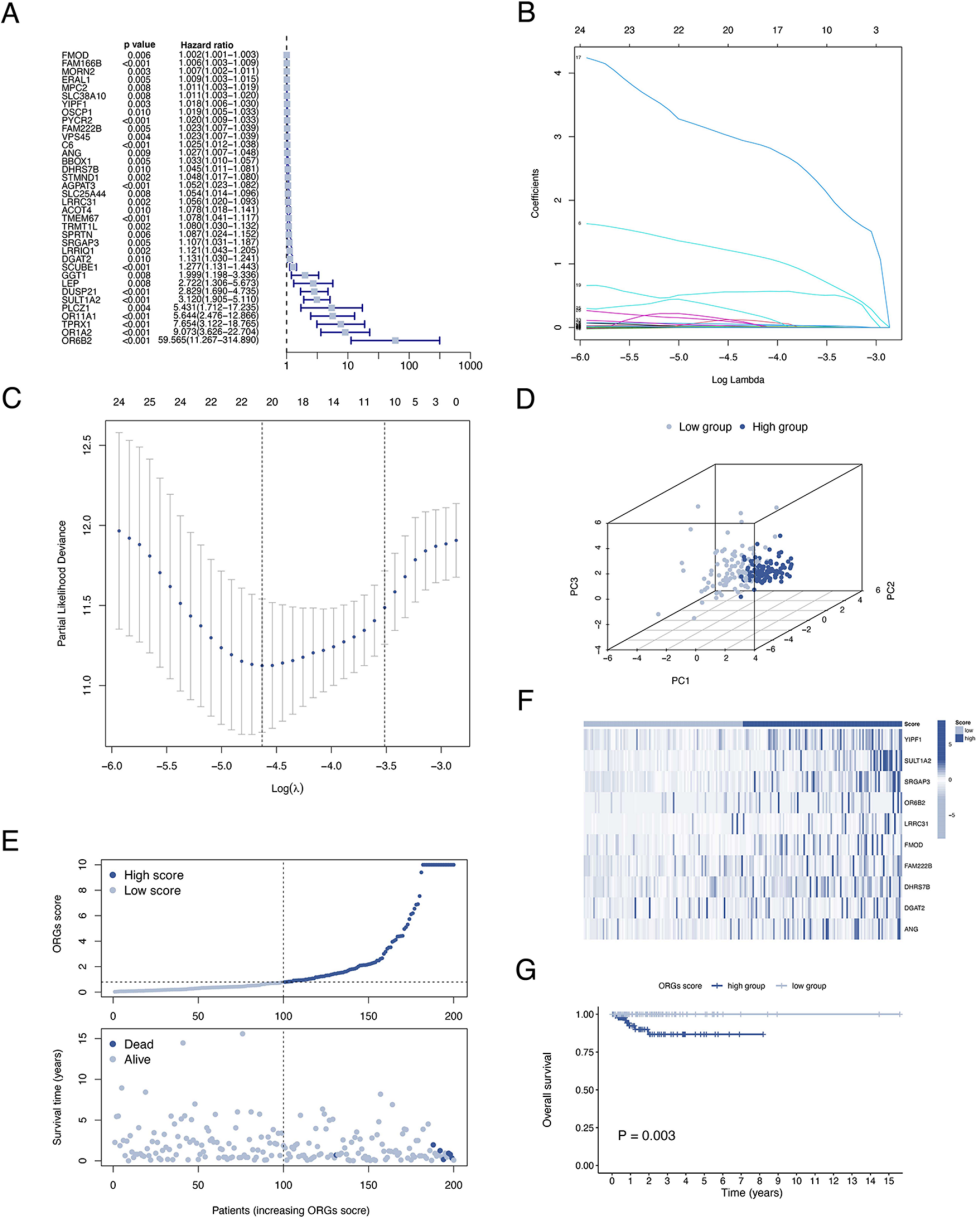
Establishment of the ORGs signature in the train cohort. (**A**) Univariate Cox regression analysis for screening prognostic ORGs. (**B,C**) LASSO regression analysis for variable selection and avoid overfitting. (**D**) PCA plot for different ORGs scoring groups. (**E**) Scatter diagram for the ORGs score and survival status of EEC patients. (**F**) Heat map for the key 10 ORGs expression with ORGs grouping. (**G**) Kaplan–Meier curves of survival difference between two groups. ORGs, obesity-related genes. LASSO, least absolute shrinkage and selection operator. PCA, principal component analysis. EEC, endometrioid endometrial cancer.

**Figure 4:**
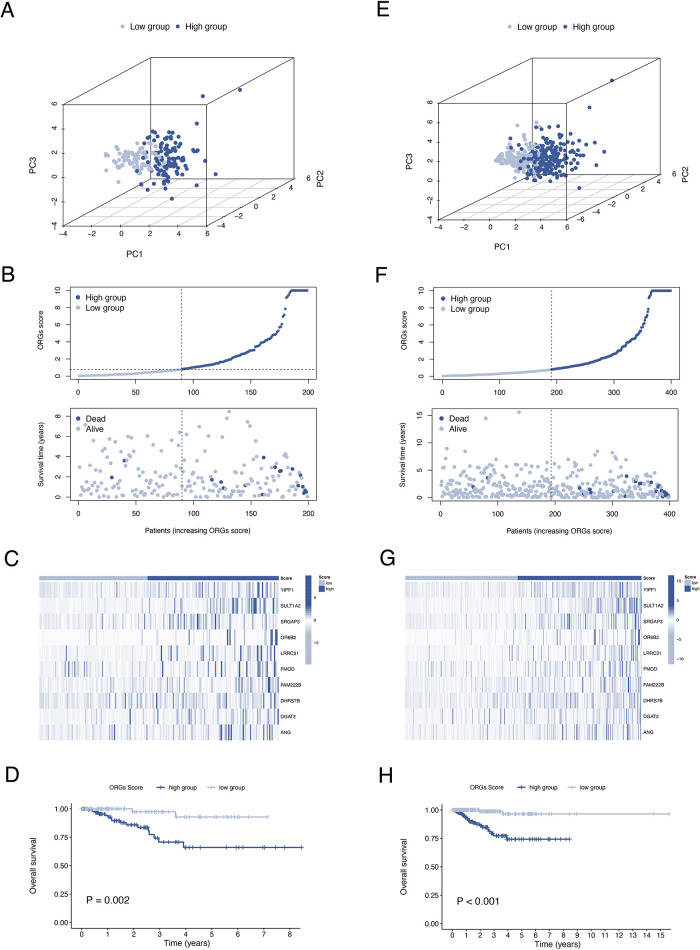
Validation of the ORGs signature in the test cohort and the entire cohort. PCA plot (**A**), scatter plot (**B**), heat map (**C**), and Kaplan–Meier curves (**D**) for the test cohort. PCA plot (**E**), scatter plot (**F**), heat map (**G**), and Kaplan–Meier curves (**H**) for the entire cohort. ORGs, obesity-related genes. PCA, principal component analysis.

A total of 399 cases from the TCGA database were included in this study and were equally divided into training and test groups. Univariate regression analysis of ORGs combined with transcriptomic data and clinical data showed that a total of 36 ORGs were significantly associated with prognosis in EEC patients (*P* < 0.001, Figure 3A). We then conducted LASSO and stepwise multivariate Cox regression for prognosis-related ORGs. Finally, 10 key ORGs were identified for the construction of the prognostic signature (Figures 3B,C). The ORGs score was calculated according to the following equation, and the training cohort was divided into high- and low-scoring groups according to the median of the ORGs score: YIPF1 × 0.017847 + SULT1A2 × 1.8463 + SRGAP3 × 0.082928 + OR6B2 × 5.6732 + LRRC31 × 0.078038 + FMOD × 0.0014647 + FAM222B × 0.033231 + DHRS7B × 0.032636 + DGAT2 × 0.10261 + ANG × 0.025124. The PCA plot illustrated the distribution of differences between the two ORGs groups (Figure 3D). The survival time and survival status of ORGs score in EEC patients were shown as scatter plots, where the survival time decreased and the number of deaths increased with increasing ORGs score (Figure 3E). The expression of key genes of ORGs signature and their correlation with ORGs groups are shown by heat map (Figure 3F). Kaplan-Meier curves demonstrated significant differences in survival between ORGs groups (*P* = 0.003, Figure 3G). Similar results were observed in the testing cohort and the entire cohort, which verified the strong and robust predictive power of ORGs signature (Figures 4A–H).

### Correlation analysis of clinical characteristics

**Figure 5:**
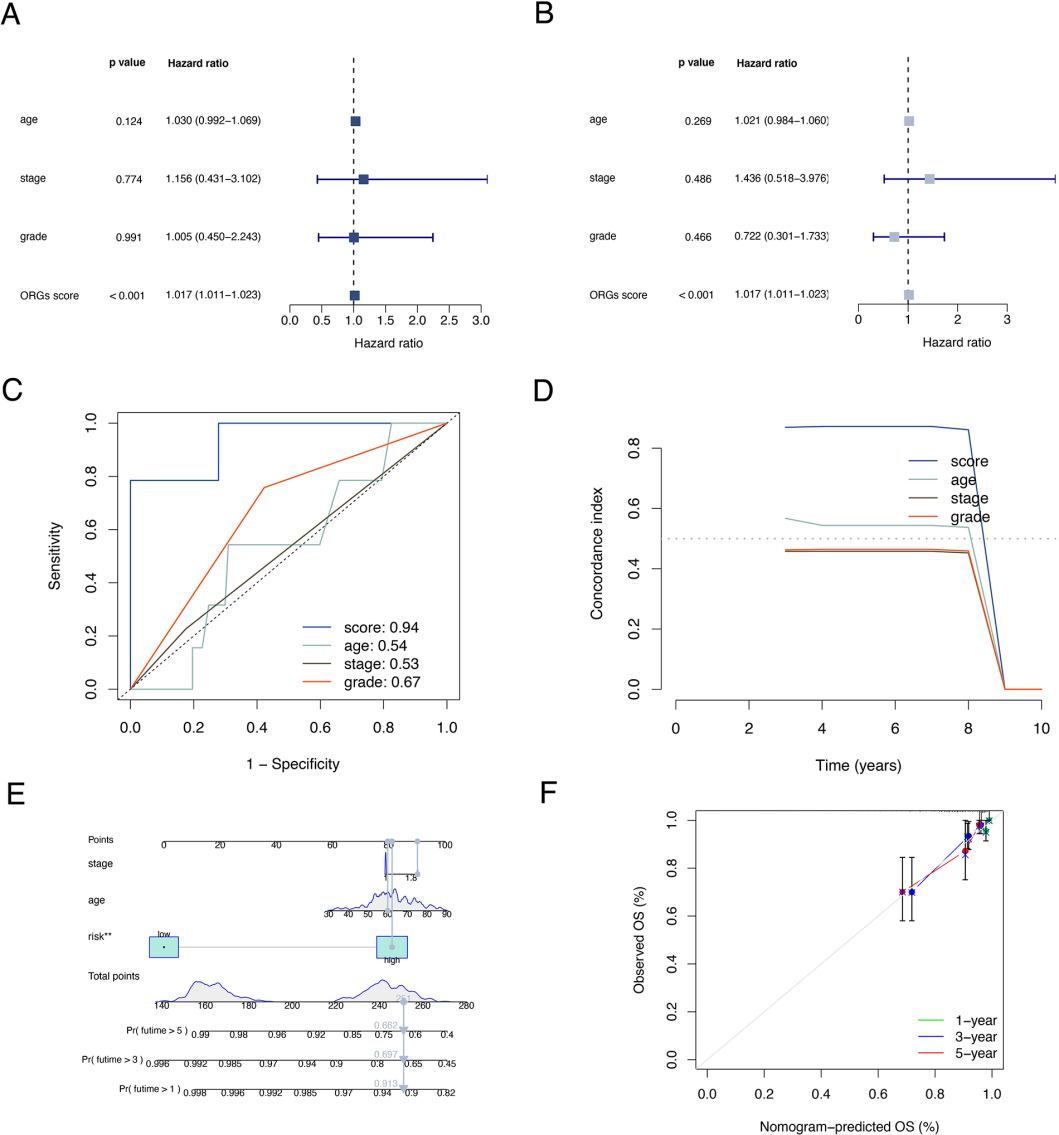
Correlation analyses of clinical features. Univariate (**A**) and multivariate (**B**) Cox regressions of ORGs score, age, tumor grade, and FIGO stage. The ROC (**C**) and C-index (**D**) of ORGs score, age, tumor grade, and FIGO stage. (**E**) The nomogram for predicting prognosis of EEC patients. (**F**) The calibration curves of the nomogram. ORGs, obesity-related genes. FIGO, the International Federation of Gynecology and Obstetrics. ROC, receiver operating characteristic curve. EEC, endometrioid endometrial cancer.

**Figure 6:**
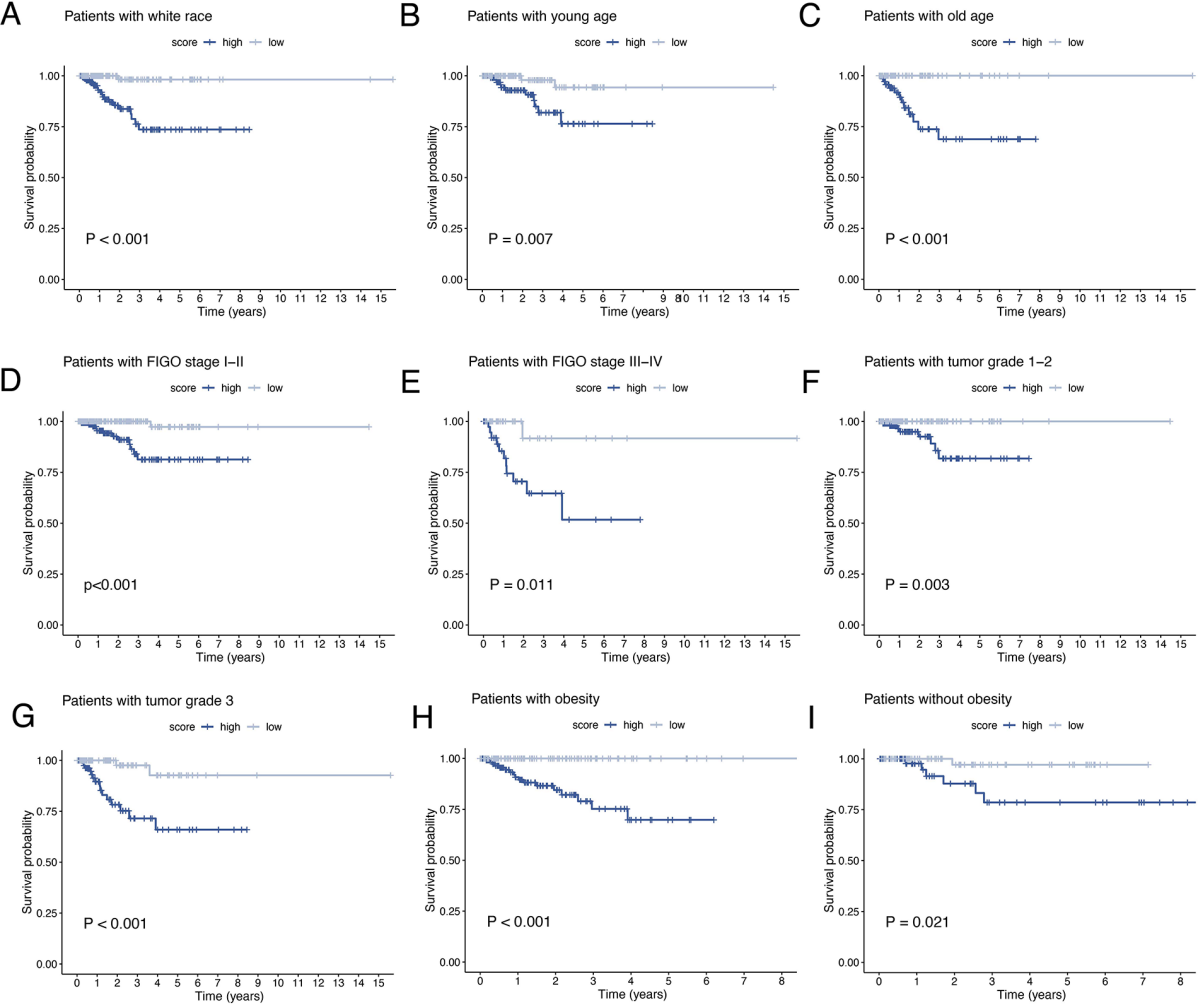
Subgroup analysis of EEC patients. Kaplan–Meier curves of patients with white race (**A**), age < 65 (**B**), age ≥ 65 (**C**), FIGO stage I–II (**D**), FIGO stage III–IV (**E**), tumor grade 1–2 (**F**), tumor grade 3 (**G**), BMI ≥ 30 (**H**), BMI < 30 (**I**). EEC, endometrioid endometrial cancer. FIGO, the International Federation of Gynecology and Obstetrics. BMI, body mass index.

The results of univariate and multivariate Cox regression analyzes demonstrated that the ORGs score was an independent risk factor that affected the prognosis of patients with EEC (Figures 5A,B). Receiver operating characteristic curve (ROC) analysis and the C index curves demonstrated the strong predictive power of the ORG score compared to other clinicopathological characteristics (Figures 5C,D). Integrating the ORGs score and relatively complete clinical characteristics including age, race, FIGO stage, and tumor stage, we constructed a nomogram to predict 1-year, 3-year, and 5-year survival rates after diagnosis in EEC patients (Figure 5E). We also plotted calibration curves to confirm the agreement between the predictions of the nomogram and actual observations (Figure 5F). To further confirm the prognostic value of ORGs signature, we performed subgroup analyzes of different clinicopathological characteristics of EEC patients in the entire cohort. The results showed that ORGs grouping was associated with the prognosis of patients with EEC among those white, of different age, different tumor grade, different FIGO stage, and different BMI (*P* < 0.05; Figures 6A–I). This suggests that the ORGs signature retains valid predictive power across subgroups of age, race, BMI, FIGO stage, and tumor grade.

### Correlation analysis of immune function

**Figure 7:**
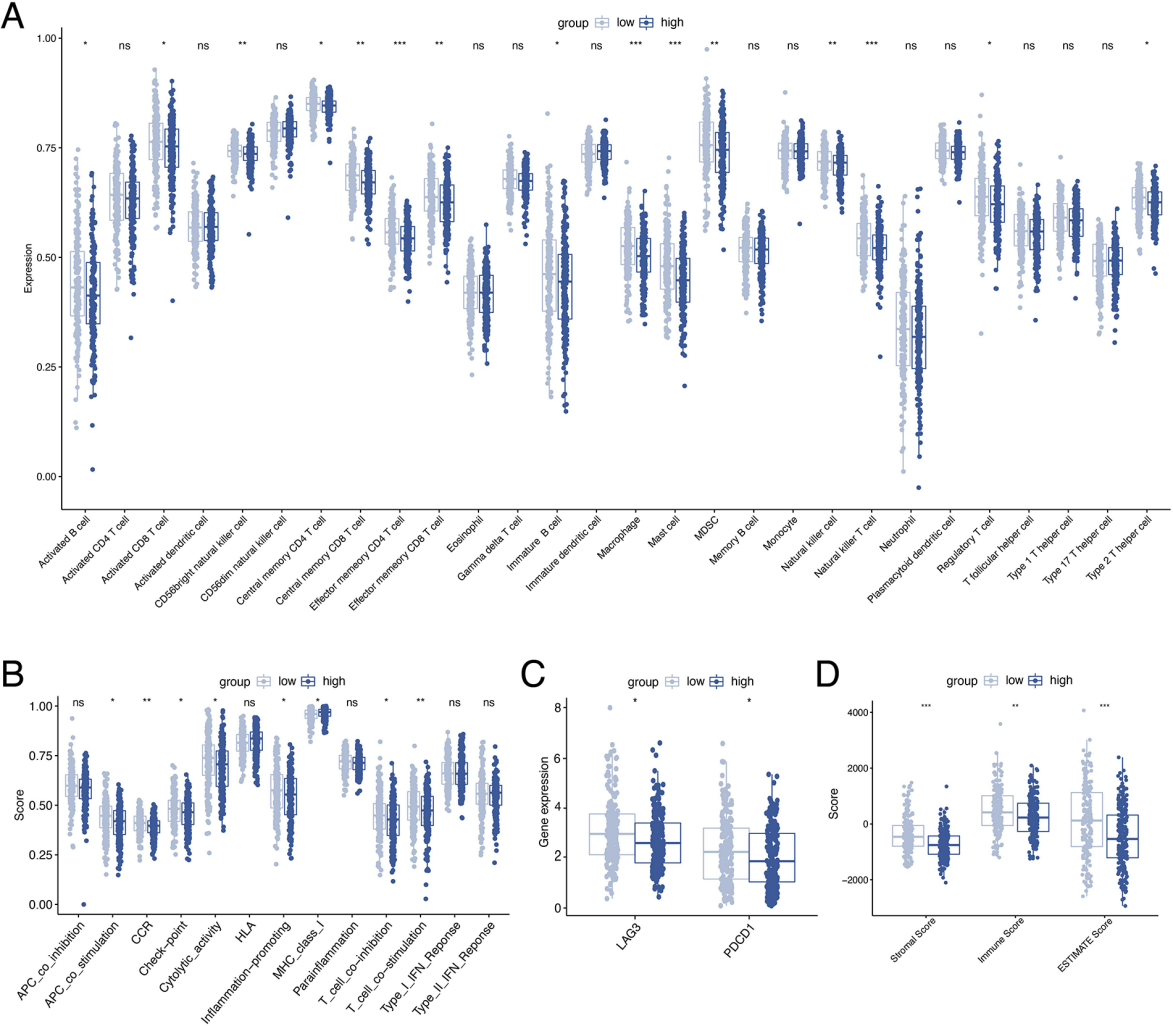
Analysis of immune activity. Comparison of the discrepancy of immune cell infiltration (**A**) and immune function (**B**) between two groups based on ssGSEA. (**C**) Differences in the expression of LAG-3 and PD-1 between the two groups. (**D**) TME analysis based on the ESTIMATE algorithm. ssGSEA, single-sample gene set enrichment analysis. TME, tumor microenvironment.

To investigate the relationship between ORGs grouping and immune status, ssGSEA was performed for each immune cell subset and functional pathway. Activated B cell, Activated CD8 T cell, CD56bright natural killer cell, Central memory CD4 T cell, Central memory CD8 T cell, Effector memory CD4 T cell, Effector memory CD8 T cell, Immature B cell, Macrophage, Mast cell, MDSC, Natural killer cell, Natural killer T cell, Regulatory T cell, and Type 2 T helper cell were significantly elevated in the low-scoring group compared to the counterpart (Figure 7A). The immune function score showed that the low-scoring group was more active in APC co stimulation, CCR, Check-point, Cytolytic activity, Inflammation-promoting, MHC class I, T cell co-inhibition, and T cell co-stimulation (Figure 7B). Furthermore, we analyzed the expression of immune checkpoint genes between the two groups and found that the immune checkpoint genes LAG3 and PD-1 were more expressed in the low-scoring group (Figure 7C). In addition, we calculated the tumor microenvironment (TME) score according to the ESTIMATE algorithm. Likewise, the results revealed a higher infiltration of stromal cells and immune cells in the low-scoring group (Figure 7D).

### Gene mutation analysis

**Figure 8:**
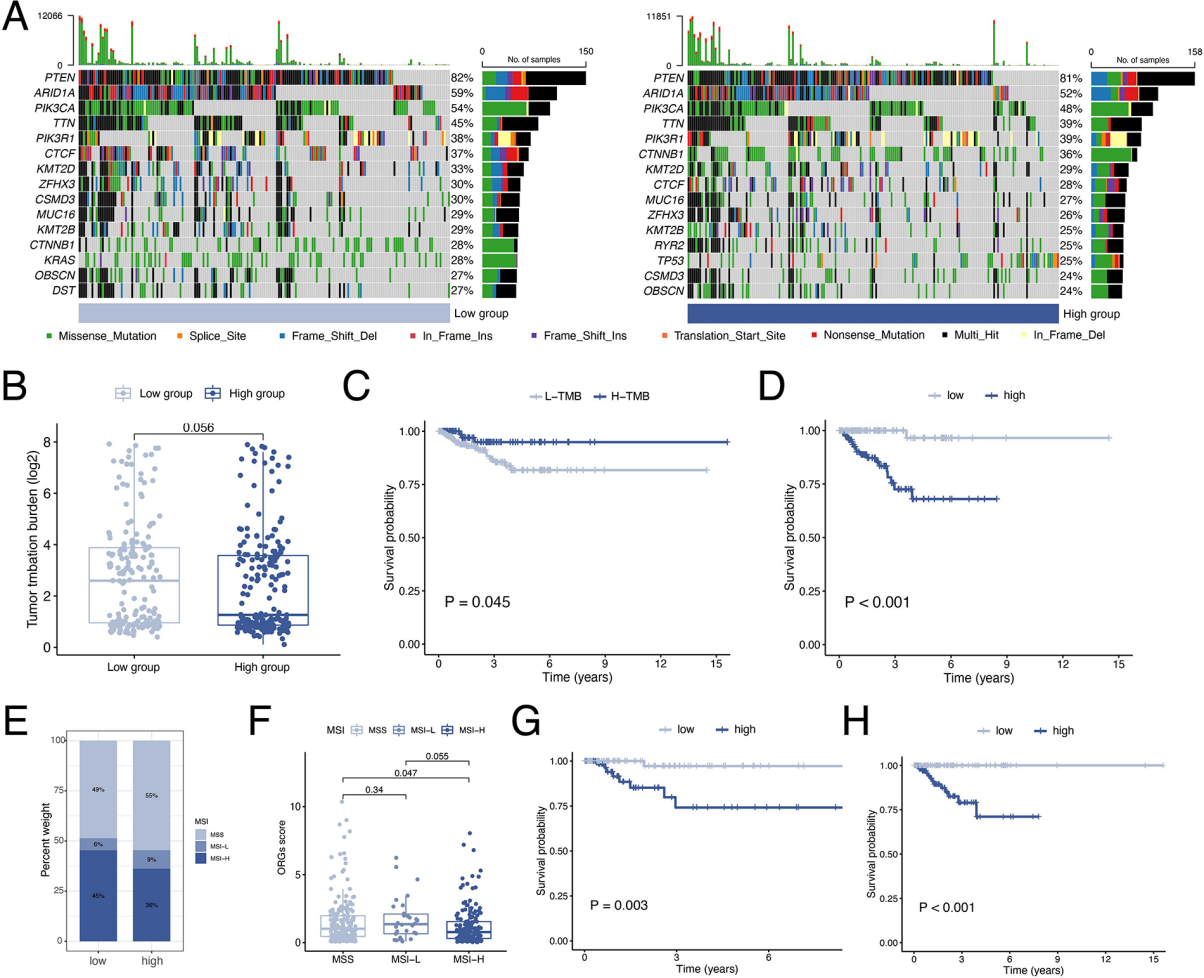
Analyses of mutation data. (**A**) Visualization of somatic mutations in different ORGs groups. (**B**) Differential analysis of TMB in ORGs scoring groups. (**C**) Kaplan–Meier curves of survival differences between the high- and low-TMB groups. (**D**) Kaplan–Meier curves of survival differences between ORGs scoring groups in low-TMB patients. (**E**) Distribution of MSI status in the different scoring groups. (**F**) Differential analysis of the ORGs score in patients with different MSI status. Kaplan–Meier curves of survival differences between ORGs scoring groups in patients with MSI-H (**G**) and MSS (**H**). ORGs, obesity-related genes. TMB, tumor mutation burden. MSI, microsatellite instability. MSI-H, high microsatellite instability. MSS, microsatellite stable.

By waterfall graphs, we visualized somatic mutations in different ORGs groups (Figure 8A). PTEN, ARID1A, PIK3CA, and TTN were mutated frequently in the EEC and more frequently in the low-scoring group. After that, we calculated the tumor mutation burden in each group and observed no significant difference in TMB levels between ORGs groups (Figure 8B). Grouped by median TMB, patients with high TMB had a better prognosis (*P* = 0.045, Figure 8C). In the subgroup with low TMB, the ORGs score had prognostic predictive value (Figure 8D). In addition, we analyze the correlation between MSI and ORGs score. The histogram illustrated the discrepancies in the distribution of MSI status in the different scoring groups. The proportion of high-frequency MSI (MSI-H) was higher in patients with low ORGs score (Figure 8E). Patients with MSI-H had lower ORGs score compared with microsatellite stability (MSS) patients (*P* = 0.047, Figure 8F). The ORGs score has prognostic value in patients with MSI-H and MSS (Figures 8G,H).

### Drug sensitive analysis

**Figure 9:**
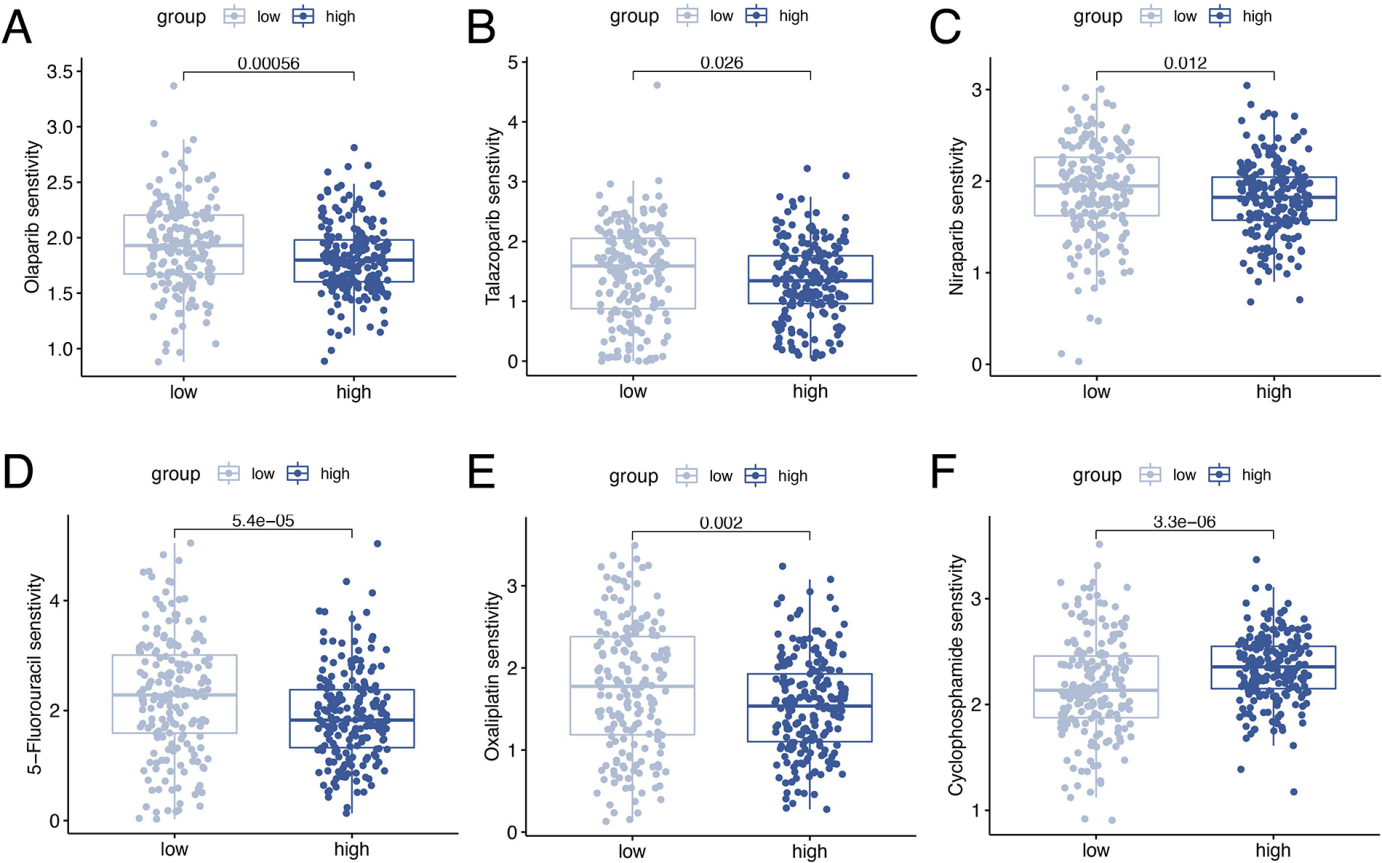
Drug sensitivity analyses. Analysis of drug sensitivity differences in olaparib (**A**), talazoparib (**B**), niraparib (**C**), 5-fluorouracil (**D**), oxaliplatin (**E**), and cyclophosphamide (**F**).

Drug sensitivity analysis revealed significant differences in ORGs scoring group among various gynecologic antitumor drugs including olaparib, talazoparib, niraparib, 5-fluorouracil, oxaliplatin, and cyclophosphamide (Figures 9A–F).

## Discussion

Unlike other reproductive system tumors that threaten women's life and health, EC is a serious disease burden as its morbidity and mortality are increasing year by year. This could be due to the combined effects of an aging population, a decrease in benign hysterectomy, and the prevalence of obesity (13). As one of the most important factors associated with the development of EC, obesity seriously affects the prognosis of patients with EC by making surgery and perioperative management more difficult and increasing the risk of comorbidities and complications (7). The current view is that EEC is the subtype most strongly associated with obesity. Analyzing the association between obesity and EEC based on gene expression data will help us to better understand potential mechanisms, select more appropriate therapeutic regimens for patients with different molecular characteristics, and improve the prognosis of EEC patients.

In this study, a gene module significantly associated with obesity was identified using WGCNA based on the GSE6008 data. The enrichment analysis of the module genes showed significant enrichment in fatty acid metabolism and biooxidation pathways. Subsequently, we constructed an ORGs signature using RNA sequencing data from EEC samples in TCGA. Subsequently, extracting RNA sequencing data from EEC samples in TCGA, we constructed a signature of ORGs including *ANG*, *SULT1A2*, *DGAT2*, *YIPF1*, *SRGAP3*, *LRRC31*, *FMOD*, *OR6B2*, *DHRS7B*, and *FAM222B*. *ANG*-encoding proteins belong to the ribonuclease A superfamily and have been widely reported to be associated with the invasion and progression of various cancers. In colorectal cancer, *ANG* cleavage produces tRNA-derived stress-induced small RNAs (tiRNAs) that promote colorectal cancer metastasis (14). Meanwhile, elevated expression of ANG was found to affect endometrial angiogenesis in hyperinsulin-treated mice (15). Integrating previous reports and bioinformatics analysis, we hypothesized that *ANG* expression is associated with obesity and involved in EC progression by affecting tumor angiogenesis. *SULT1A2* encodes phenol sulfotransferases involved in hormone metabolism and has been reported to be associated with the prognosis of patients with HER2-positive breast cancer (16). Also, as estrogen-dependent tumors, the development of EEC may be influenced by *SULT1A2* expression. *DGAT2* encodes a key enzyme that catalyzes the synthesis of triglycerides and has been reported to be involved in the reprogramming of lipid metabolism in tumor cells, driving tumor progression (17). Similarly, the correlation between *YIPF1*, *SRGAP3*, *LRRC31*, *FMOD* and various tumors has been reported in the literature (18–21). However, the correlation between *OR6B2*, *DHRS7B*, *FAM222B*, and tumors has been little explored. In future studies, we should experimentally investigate their role in the development of EEC.

According to the ORGs score, EEC patients were divided into two groups. PCA and survival analysis demonstrated the differences in the distribution between the two groups and the prognostic value of the ORGs signature. We then applied Cox regression and ROC analysis to clarify the value of the ORGs score as an independent risk factor for EEC patients. Subgroup analysis demonstrated that ORGs score-based grouping maintained considerable survival predictive power in different clinical subgroups. Integrating age, FIGO stage, tumor grade, and ORGs score, we further developed a prognostic nomogram to stratify the prognosis of EEC patients to support clinical practice.

By analyzing somatic mutation data, we found discrepancies in gene mutation frequencies between ORGs groups. The frequency of *ARID1A* mutations was significantly higher in the low-scoring group. It was shown that *ARID1A* protein expression deletion occurred more frequently in high-grade EEC and was associated with activation of the PI3K/AKT pathway (22, 23). The higher frequency of *CTNNB1* mutations in the high-scoring group, with reduced expression of its encoded protein *β*-catenin, was involved with disease progression and associated with poor prognosis (24, 25). The TMB was calculated using mutation data and found that the ORGs score still had the ability to stratify the prognosis in the low-TMB group.

Currently, immunotherapy for EC is a popular concern among gynecological oncologists. Immune checkpoint blockers (ICBs) activate the immune system to kill tumors by relieving T-cell suppression through binding to their targets (26). Based on the findings of Keynote 028 and Keynote 158, the current view is that ICBs are beneficial for recurrent or metastatic EC patients with TMB-H, MSI-H and PD-1/PD-L1-positive (27–29). This study revealed differences in immune cell infiltration, PD-1 expression, and MSI status between the ORGs groups. Innate and specific immune cells were more infiltrated in the low-scoring group, and PD-1 expression and MSI-H proportion were higher. Given the survival discrepancies between different ORGs groups in each MSI subgroup, we speculate that integrating ORGs score, MSI status, TMB levels, and immune checkpoint gene expression might allow further screening of EEC patients and precise targeting of immunotherapy benefit populations.

Considering the promising results of poly (ADP-ribose) polymerase inhibitors (PARPi) in the maintenance treatment of ovarian cancer, numerous studies have converged on the possibility of PARPi application in the treatment of EC (30, 31). The results of our sensitivity analysis for commonly used gynecologic antineoplastic agents showed that the ORGs groups differed in drug sensitivity for a variety of PARPi, including olaparib, niraparib and talazoparib. The possibility of ORGs score for screening potential PARPi beneficiaries and the mechanism of correlation between ORGs and PARPi still needs to be clarified by further studies.

There are unavoidable limitations to this study. The data used to construct and validate the model were obtained from retrospective public databases and the conclusions of this study should be further validated by prospective data. In addition, the hypotheses established based on the results of immune, mutation and drug sensitivity analyses need to be further confirmed by functional experiments.

## Conclusion

In the present study, the ORGs signature was established and analyzed in terms of clinical characteristics, mutation data, immune correlation and drug sensitivity, which found new biomarkers for exploring the underlying mechanisms of obesity and EEC and provided new insights into the precise treatment of EEC patients.

## Data Availability

The original contributions presented in the study are included in the article/Supplementary Material, further inquiries can be directed to the corresponding author/s.
